# Increased Readmission Rates in Younger Male Patients Due to Suicidal Risk in Newly Diagnosed Depressive Disorders After Initiation of Serotonin Reuptake Inhibitors

**DOI:** 10.7759/cureus.31987

**Published:** 2022-11-28

**Authors:** Ivan E Pagan Colon, Jessica Kroin, Shivani Kaushal, Sara Khan, Clara L Alvarez Villalba

**Affiliations:** 1 Psychiatry, HCA Florida Aventura Hospital, Aventura, USA; 2 Psychiatry, Dr. Kiran C. Patel College of Allopathic Medicine, Nova Southeastern University, Davie, USA

**Keywords:** hospital readmission rate, child and adolescent psychiatry, suicide and depression, selective serotonin reuptake inhibitor (ssri), age and depression

## Abstract

Background

Depressive disorders have a prevalence of 322 million people worldwide and are a leading cause of morbidity. These disorders can affect individuals of all ages and can present over time. Due to the diversity in the presentation of depressive disorders, vigilance towards depressive disorders can lead to more timely and effective treatment. Serotonin Selective Reuptake Inhibitors (SSRIs) and Serotonin Norepinephrine Reuptake Inhibitors (SNRIs) are the first lines of treatment for these disorders. Moreover, the United States Food and Drug Administration (FDA) issued a black-box warning for several antidepressants, stating an increased risk of suicidality in individuals under 25 years old. However, the placement of this black-box warning has been controversial. In this study, the authors aim to investigate if there is a relationship between the use of SSRI or SNRI on patients with newly diagnosed depressive disorder and hospital readmission due to suicide-related events.

Methods

For this retrospective cohort study, de-identified data were obtained from the HCA Healthcare database by searching for patients newly diagnosed with depressive disorders and started on SSRIs or SNRIs. Patient data were evaluated for readmissions due to suicide-related events within 90 days of discharge from the hospital and establishing their initial SSRI/SNRI prescription.

Results

After data was obtained and evaluated via statistical analysis, the variables with statistical significance were: age (p-value = 0.0164) and sex (p-value = 0.0150). These two were significantly associated with the rate of readmission: younger and male patients had an increased risk of readmission due to suicide-related events within 90 days of discharge after starting SSRI, or SNRI, to treat depressive disorders.

Conclusion

These results support the importance of monitoring patients started on SSRI or SNRI, with particularly careful consideration in depressed young male patients.

## Introduction

Depressive disorders are characterized by negative emotions severe or persistent enough to interfere with function, often accompanied by decreased interest or pleasure in activities [1]. These disorders have a prevalence of 322 million people worldwide, with 15% of this population living in the Americas [2]. Depressive disorders were among the leading causes of years lived with disability in both sexes in 2017, and rates of depressive disorders increased by 18.4% between 2005-2015 [2,3]. These statistics have compounded since the beginning of the COVID-19 pandemic in 2020, with elevated depressive symptoms rising from 27.8% in 2020 to 32.8% in 2021 in the U.S. [4]. Also, the prevalence of clinically elevated depression and anxiety in children and adolescents rose significantly and was higher in studies collected later in the pandemic [4]. With these rising rates of mood disorders, it is increasingly important to continue evaluating the efficacy and potential adverse effects of these patients’ treatments. Serotonin Selective Reuptake Inhibitors (SSRIs) and Serotonin Norepinephrine Reuptake Inhibitors (SNRIs) are the standard of care for patients with depressive disorders [5]. Ambiguous data exists regarding the use of these medications in populations younger than 24 years old. Numerous SSRIs and SNRIs have shown good tolerability and efficacy compared to placebo in treating pediatric depressive disorders. However, there remains doubt whether these medications’ benefits outweigh the risks [6]. Of the medications proven beneficial for treating these disorders in young people, only fluoxetine is FDA-approved for treating depression in children. At the same time, escitalopram is also approved for use in adolescents with depression [7]. 

One of the main adverse effects associated with SSRIs is their FDA black-box warning in 2004 and SNRI in 2006 due to studies suggesting that these medications were associated with an increased risk of suicidality in patients younger than 24 years old [6,8]. This black-box warning has been controversial, with critics stating that it unduly detracts from the benefits of SSRIs based on a likely non-causal relationship, leading to less utilization of the effective, first-line treatment for depressive disorders. Additionally, the warning has been criticized for not accomplishing its desired effect: in the years following the FDA warning, antidepressant prescribing decreased for pediatric depression, but without a compensatory increase in alternatives such as psychotherapy [9]. Others argue that the empirical evidence to date justifies the warning and the caution surrounding SSRIs [10]. Current data remains ambiguous: pooled adverse event data from an FDA analysis showed a higher risk of suicidal ideation or behavior with antidepressants compared to placebo. Other studies show no relationship or even decreased rates of suicidality following SSRI initiation [11,12]. With the rising rates of depressive symptoms and anxiety compounded by the COVID-19 pandemic, continuous evaluation of mental health treatments over which there is still dispute, such as SSRI and SNRI, is needed to ensure that therapies continue to be as effective and safe as possible. This study aims to investigate if there is any relationship between patients starting SSRIs or SNRIs for newly diagnosed depressive disorders and hospital readmissions due to suicide-related events. 

## Materials and methods

The East Florida Division of HCA corporate database of de-identified patient information was queried for patients newly diagnosed with depressive disorders who did not have a history of taking SSRIs or SNRIs. Inclusion criteria for this study were: Patients with Major Depressive Disorder (MDD) single episode (including specifiers mild, moderate and severe), MDD unspecified, other specified depressive disorder, depressive disorder due to another medical condition with depressive features, unspecified depressive disorders, dysthymia, depressive disorder due to another medical condition with a major depressive-like episode, depressive disorder due to another medical condition with mixed features, and premenstrual dysphoric disorder were included in the data query. The exclusion criteria for this study were patients diagnosed with MDD with recurrent episodes treated with SSRI or SNRI or had a prior history of taking SSRIs or SNRIs for any condition. The statistical analysis examined the relationship between the readmission rate due to suicide-related events and the predictor variables of specific SSRI or SNRI medication utilized, age, and sex. The data were analyzed by applying logistic regression to a model that included 90- day readmission rates for suicide-related events. Odds ratios were calculated to analyze the potential effect of predictor variables on readmission rate outcomes.

## Results

A total of 2,585 patients were included in the data analysis after applying inclusion and exclusion criteria. Ages ranged from 7-90 years old, and 66% of the patients were female. 85% of the patients reported their race as White, 9% reported their race as Black, and 6% specified their race as “Other” (race not White or African American). 

Logistic regression of the available data reported that the age factor reached statistical significance in association with the readmission rate. The youngest patient in the study was seven years old, and the oldest patient was described as “90+” within the system (table 1). The odds of experiencing a 90-day readmission for suicidal ideation are more likely for younger patients than older patients (p-value = 0.0164; Odds Ratio: 0.979; 95% C.I.: [0.963-0.996]).

**Table 1:** Age for Patients in Total Population

Variable	Mean	Median	Mode	Std. Dev.	Minimum	Maximum	N
Age	54.89	57	59	19.95941	7	90	2585

Regarding gender, male patients exhibited a significantly higher risk for 90-day readmission due to suicidal ideation than female patients (p-value=0.0150; Odd Ratio: 2.002; 95% C.I.: [1.144-3.503]). 

Medications evaluated for this project were SSRIs and SNRIs: Fluoxetine, Paroxetine, Fluvoxamine, Escitalopram, Sertraline, Citalopram, Venlafaxine, and Duloxetine. None of these medications reached statistical significance in association with readmission within 90 days for suicidal ideation. Similar findings were obtained when comparing factors such as marital status and race, neither of which reached statistical significance regarding readmission. 

## Discussion

Age

**Figure 1:**
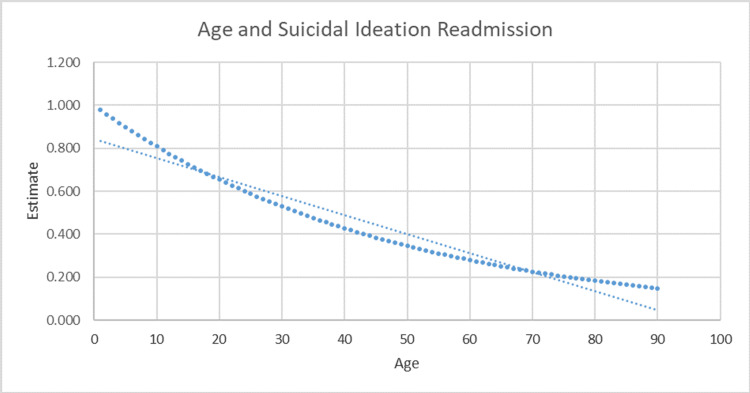
Age and Suicidal Ideation Readmission

The results in this study related to age and readmission due to suicide-related events support further monitoring of young male patients when initiated on SSRI or SNRI for depressive disorders. Investigation into the impact of SSRIs on the adolescent brain has continued to be controversial in treating depressive disorders. Our study results show that age significantly affects readmission rates following the initiation of SSRI: the odds of experiencing readmission due to suicidal ideation within 90 days of hospital discharge are higher for younger patients than for older patients (Figure 1). These results appear to support the FDA black-box warning against the use of SSRIs in patients younger than 24 years old, issued due to an increased subsequent risk of suicidal thoughts in these patients. This study’s results of increased hospitalization in younger patients are outcomes that would be expected of the increased suicidal ideation in children and adolescents seen in the placebo-controlled trials that led to issuing of the black-box warning. This consistency is a significant finding among abundant studies and clinical opinions countering the black-box warning [5,8,12].

However, comparisons to previous studies should be made cautiously, as the criteria for suicidality differ amongst different studies. For example, one limitation of our study was the lack of a rating scale to assess suicidal ideation; such scales are the most commonly utilized measures in the current literature on suicidality following SSRI initiation. One example of such a measure is The Columbia Classification Algorithm of Suicide (C-CASA), which categorizes information in patients’ medical records (i.e., suicide, suicidal attempts, preparatory acts, and ideation) as suicidality. Sorenson et al. reported a decrease in the risk of suicidality among patients under 17 in the six weeks following the initiation of an SSRI [11]. Naslund et al. reported a net neutral effect of SSRIs on the suicidality item of the Hamilton Rating Scale for Depression (HRSD) in young adults (ages 18-24) over six weeks following SSRI initiation [5].

Again, comparisons of our study with such previous studies are limited, as the length of follow-up and modes of assessing suicidality were different. However, readmission rates due to suicidal ideation could be considered a more objective measure of the risk of suicidality, and suicidality ratings may not have captured the modifying effects of any intervening hospitalizations. Results in this study should also be considered in the context of clinical risk versus benefit analysis, particularly for the specific patients being treated. In adolescent patients, including some who were suicidal, SSRIs reduced self-reported suicidality and prevented relapses of major depression [13,14]. However, it was found that after the FDA issued its advisory, monitoring of potential side effects in depressed patients taking antidepressants did not change, as the frequency of provider contact with these patients did not increase [15]. Thus, it is essential to highlight the need to expand monitoring systems and further research regarding side effects due to the use of SSRIs. It is recommended that the data on the potential adverse effects of antidepressants be incorporated into clinical practice to treat patients with depressive disorders effectively. 

Another primary concern in treating adolescent depression is the less-known effects of SSRIs on the developing brain of young patients. For these potential effects, many reviews have concluded that little evidence exists to suggest that SSRIs put the adolescent brain at developmental risk [16]. However, these conclusions are based on clinical outcomes, and further research is needed to elucidate specific age-dependent effects of SSRIs on short- and long-term brain development. 

Gender 

**Figure 2:**
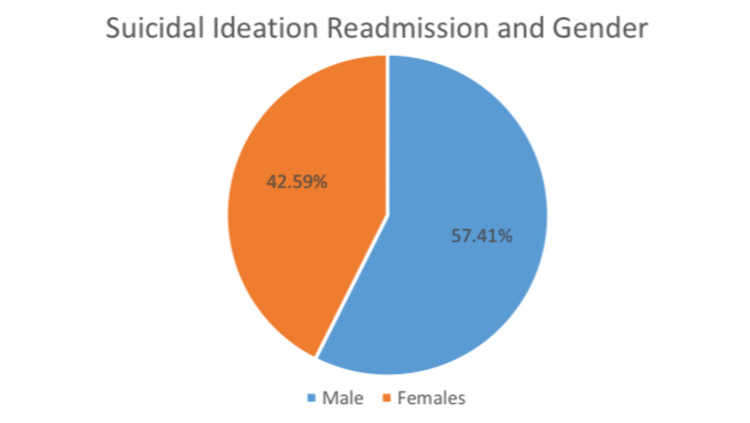
Suicidal Ideation Readmission and Gender

Similar to the results in this investigation, one retrospective study found that the male gender and the presence of suicidal ideation on admission are predictive of higher rates of psychiatric readmission within 90 days [17] (Figure 2). It remains unclear why males with suicidal ideation have higher readmission rates than females with suicidal ideation; however, several factors may be at play. A retrospective chart reviewing demographic factors about voluntary psychiatric admissions found that males were significantly more likely to have previous psychiatric admissions, test positive for substances, and be homeless, unemployed, and uninsured [18]. Although the chart review excluded involuntary psychiatric admissions, the results suggest that social factors may contribute to higher psychiatric readmission rates among males. In addition, the higher readmission rates may also be explained by possible gender differences in the severity of depressive symptoms. A cross-national study on gender differences in suicide intent found that males were likelier to demonstrate suicidal behaviors with clear intent to die. At the same time, females were more likely to exhibit parasuicidal behaviors, which are suicidal behaviors where the primary motive is not to die but to escape or manipulate a situation [19]. These results support the well-established ‘gender paradox in suicidal behavior’, based on statistics indicating that males are more likely to complete suicide. At the same time, females are more likely to engage in nonfatal suicidal behavior. This difference may suggest that the progression of suicidality occurs more quickly in males than in females [20]. 

Medication

A statistically significant association did not exist between a specific SSRI or SNRIs and the risk for readmission due to suicide-related events within 90 days. This finding requires further investigation, particularly about the factors considered in the FDA approval of some antidepressant drugs over others in young patients with depressive disorders. Of the antidepressants, only fluoxetine and escitalopram are FDA-approved for treating depression in children and adolescents based on clinical trial data. While additional research is needed regarding readmission rates, multiple trials have indicated that several SSRIs, including fluoxetine and escitalopram, demonstrated treatment gains within the first two weeks among pediatric patients with MDD [21]. This early onset of effect is similar to that seen in adults with MDD on SSRIs. It may result in lower readmission rates among pediatric patients treated with SSRIs versus other antidepressants. 

Limitations

The data obtained did not encompass information about the social environment, employment, home and family dynamics, participation in behavioral therapy, socioeconomic status, medical history, and other factors that could have played a role in patients’ emotional states and overall mental health status. Further studies should investigate such factors to account for the significant impact of psychosocial influences on emotional well-being and how these aspects may work in tandem with the effects of antidepressant medications. Another limitation was related to the potential of a patient having a dual diagnosis and being treated with SSRIs or SNRIs. While requesting the data for this study, there was limited information regarding the specificity related to the suicidal action (planning, attempt, ideation) for which the patient was readmitted. Such information, taken with the currently available data about age and gender, can allow clinicians to consider even more specific relationships between the benefits versus risk of SSRIs and SNRIs and their impacts on patients.

## Conclusions

Based on the results obtained in this study, there is an increased risk for readmission due to suicide-related events within 90 days for younger patients after starting SSRI or SNRI treatment for depressive disorders. This is per the black-box warning placed on these medications. An increased risk for readmission due to suicide-related events was also seen in males compared to females. Thus, while the controversy regarding using SSRI and SNRI in the younger population (patients 24 years old or less) remains, this supports the recommendation that clinicians should monitor and counsel their patients. Specifically, these results support the importance of monitoring all patients on SSRI or SNRI, with the additional consideration of caution in depressed young male patients being treated for depressive disorders.
